# Effect of Aqueous Extract of *Embelica officinalis* on Selenite Induced Cataract in Rats 

**Published:** 2010

**Authors:** Nair Kavitha Nair, Kirti Patel, Tejal Gandhi

**Affiliations:** *Anand Pharmacy College, Anand, Gujarat, India.*

**Keywords:** Cataract, *Embelica officinalis*, Selenite, Ascorbic acid, Glutathione, Malondialdehyde

## Abstract

Cataract is clouding of the eye lens that reduces the amount of incoming light and results in deteriorating vision. Blindness is thought to reach 75 million by 2020. Of these, unoperated cataract may be expected to account for at least 35 million. Thus, the burden of cataract is increasing remorselessly. *Embelica officinalis *is reported to have a very good antioxidant property and thus we hypothesized that it could be a good candidate in treatment of cataract. Hence, the aim of this study was to investigate the effect of aqueous extract of *Embelica officinalis *on selenite induced cataract in rats.

For the purpose of this study, cataract was induced in young suckling (on the 10^th ^day of life) albino wistar rats using sodium selenite (a single dose of sodium selenite; 20μM/kg; subcutaneously). After induction of cataract, the test drug (*Embelica Officinalis) *and the reference standard (ascorbic acid) were administered orally for 18 days. The progression or disappearance of cataract was observed with the help of an ophthalmoscope (OM-18, Takagi resolution 1.6). At the end of this study the alterations in the levels of total protein, soluble protein, reduced glutathione and malondialdehyde were estimated in the lens homogenate.

Results showed that treatment with *Embelica officinalis, *as well as ascorbic acid, produced a significant decrease (p < 0.05) in malondialdehyde and a simultaneous increase in lens glutathione levels (p < 0.05). The malondialdehyde content was decreased by 48% in animals treated with *Embelica officinalis*. Similarly, lens glutathione was increased by 82.5% in animals treated with *Embelica officinalis*. There was also a significant (p < 0.05) increase in protein content (total protein = 59.36% and soluble protein = 105.78%) in animals treated with *Embelica officinalis, *indicating improvement in cataractogenic condition in the selenite induced cataract model. At the end of the treatment, disappearance of cataract was observed in test and standard treated animals.

In conclusion, it could be said that aqueous extract of *Embelica officinalis *delayed the progression of cataract in sodium selenite induced cataractogenic rats.

## Introduction

Cataract is clouding of the eye lens that reduces the amount of incoming light and results in deterioration of the vision. Cataract remains the leading cause of visual disability and blindness all over the globe (1, 2), which makes up at least 50% of blindness in most developing countries (3). Blindness is thought to reach 75 million by 2020. Of these, unoperated cataract may be expected to account for at least 35 million. This figure is equivalent to the combined present total populations of Australia, New Zealand, Sweden and Denmark. Thus, the burden of cataract is increasing remorselessly (3). Senile cataract, also called “age related cataract,„ is the commonest type of cataract affecting equally persons of either sex usually above the age of 50 years. Increased oxidative stress is reported to be the cause for such a cataract. The only treatment presently available is surgery. Experimental cataract, correlating senile cataract, can be induced by subcutaneous injection of sodium selenite to suckling rats. Several biochemical processes occur during production of selenite cataract. These include altered epithelial metabolism, calcium accumulation, calpain-induced proteolysis, crystalline precipitation, phase transition, and cytoskeletal loss. A number of important changes in metabolism have been documented in lens epithelium during the formation of selenite cataract; usually well before any visible opacity. These include suppression of mitosis and entry of epithelial cells into prophase, nuclear fragmentation (4), decreased rate of epithelial cell differentiation (5), decreased synthesis and increased damage of DNA (6), and loss of calcium haemostasis (7). It has been hypothesized that these early changes in lens epithelium may result from oxidative damage caused by selenite, possibly due to critical sulfhydryl groups on molecules such as calcium ATPase or ion channels. Loss of calcium haemostasis could be prevented by antioxidants. 

Preparations containing *Embelica officinalis *are widely used for their preventive, curative, and health restorative properties. It possesses very good hypocholesterolemic (8), anticarcinogenic (9), antidiabetic (10), and antioxidant properties (10-13). *Emblica officinalis *is a rich source of vitamin C (ascorbic acid), and most of its biological actions may be due to vitamin C. Nevertheless, the reported vitamin C content in *Embelica officinalis *varies from negligible to 0.7% (14). 

Presently, the only treatment available for cataract is surgery and thus the present study was aimed to investigate the effect of *Embelica officinalis, *a herbal drug having antioxidant activity, in treatment and prevention of maturation of cataract. A prevention or delay through such an applications in human will be a significant achievement.

Hence, the objective of the present study was a preliminary investigation into the effect of *Embelica officinalis *in selenite induced cataract.

## Experimental

*Identification and collection of agents*

*(I) Embelica officinalis *was collected from the field of nearby region and dried in shade. It was authentified by the Bioscience Department, Sardar Patel University and a specimen voucher was deposited. Powdered plant was prepared and stored in an air-tight container. An aqueous extract was prepared for the purpose of study, and administered orally (p.o.).

(II) Sodium selenite was procured from Loba Chemie Pvt. Ltd., (Mumbai, India) and administered subcutaneously (s.c.). 

(III) L-ascorbic acid was procured from Astron Chemicals Pvt. Ltd., (Mumbai, India) and Administersd p.o. 

*Estimation of ascorbic acid in Embelica officinalis*

Aqueous extract of *Emblica officinalis *was estimated for the content of ascorbic acid, as per the method described in Indian Pharmacopoeia (15). 

*Selection of animals*

All animals were housed at ambient temperature (25 ± 10°C) and relative humidity (55 ± 5%), and a 12h/12h light/dark cycle. Animals had free access to standard pellet diet and water given *ad libitum*. The experimental protocol was approved by the Institutional Animal Ethical Committee, as per the guidance of committee for the purpose of Control and Supervision of Experiments on Animals (CPCSEA), Ministry of Social Justice and Empowerment, Government of India (Protocol number project 5008, dated 10th of January 2006). 

*Induction of cataract*

Cataract was induced experimentally by selenite treatment. Thirty suckling wistar rats (9 days old pups) of either sex were divided into five groups, with 6 animals in each group. On the 10^th^ day of life, when they were in an absolute nutritive dependence from the mother, a single dose of sodium selenite (20 μM/kg) was administered s.c. to all the groups except for the normal control group (group l) (16).Groups I and group II (cataract control) animals received food and water *ad libitum *throughout the study period. Group III (test) and group lV (standard) received aqueous extract of *Embelica officinalis *(equivalent to 26.19 mg/kg ascorbic acid) and ascorbic acid (26.19 mg/kg), once daily orally, respectively. 

The treatment was continued for 18 days after the initiation of cataract. The eyes of the animals were observed for the progression/ disappearance of the cataract. All the animals were sacrificed at the end of study, to isolate the lens and lens homogenate was prepared and used to estimate various biochemical parameters. Total protein and soluble protein contents were estimated, using the Lowry *et al. *(17) method, in the lens homogenate. Anti-oxidative properties were evaluated by estimating the glutathione (18) and malondialdehyde (19) levels. 

*Estimation of Malondialdehyde in lens (MDA)*

MDA estimation was performed using the method described by Ohkawva *et al *(19). Briefly, to 0.2 mL of lens homogenate, 0.2 mL of 4% sodium dodecyl suphate, 1.5 mL of 20% acetic acid (prepared in 0.27 M HCl) and 1.5 mL of 0.5% thiobarbituric acid were added. The mixture was heated for 60 min at 95°C in a temperature controlled water bath to give a pink color. The mixture was then centrifuged at 3500 rpm for 10 min. Finally, absorbance of the supernatant layer was read spectrophotometrically at 532 nm. 

*Statistical analysis*

Statistical analysis of various physical and biochemical parameters were carried out, using the one way analysis of variance (ANOVA),with p **< **0.05 considered as a significant difference.

## Results

*Effect of Embelica officinalis on catract progression*

*Embelica officinalis *extract was found to have 7.5 mg of vitamin C (0.75%).

**Figure 1:**
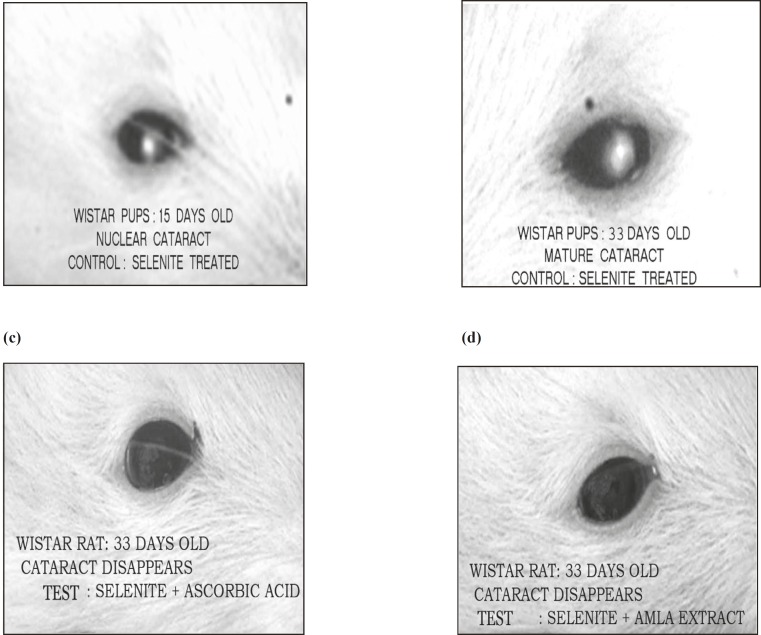
Effect of *Embelica officinalis *extract, selenite and ascorbic acid on cantaract progression.

Presence of cataract in the sucklings was visible by the naked eye after the opening of their eyes, i.e. on the 15^th^ day of life in groups II, III and IV.on the 15^th^ day of their life cataract was seen to turn yellowish –white, localized in the center of the lens, termed as nuclear cataract (Figure 1a). The cataract progressed to mature catarct in the selenite control group on day 33 after birth (Figure 1b). On the contrary, cataract was found to be disappeared in the ascorbic acid (Figure 1c) and *Embelica officinalis *(Figure 1d) treated animals. 

*Effect of Embelica officinalis on lens protein content*

Selenite injection in animals, caused a decrease in the amounts of total protein and soluble protein (Table 1). 

**Table 1:** Effect of *Embelica officinalis *on total protein, soluble protein, malondialdehyde level and reduced glutathione levels in selenite induced cataract. All the values are expressed in terms of mean ± SEM; n = 6 in each group

Parameter	Normal control	Selenite control	*Embelica officinalis * (26.19 mg/kg)	Ascorbic acid (26.19 mg/kg)
Total protein (μg/mL of tissue homogenate)	2900.0 ± 89.5	1528.0 ± 303.3^**^	2435.0 ± 231.9 ^#^	2313.0 ± 223.7
Soluble protein (μg/mL of tissue homogenate)	2773.0 ± 99.6	1140.0 ± 240.9^**^	2346.0 ± 217.3^#^	1844.0 ± 72.23^#^
Malondialdehyde (μg/mL of tissue homogenate)	0.1996 ± 0.0095	0.3901 ± 0.0697^*^	0.1901 ± 0.0231^#^	0.2398 ± .01864
Glutathione (μg/mL of tissue homogenate)	9.972 ± 0.203	8.153 ± 1.264^*^	14.880 ± 1.483	10.350 ± 0.644

Significantly different, as compared to the normal control group (p < 0.05)

**Significantly different, as compared to the normal control group (p < 0.001)

# Significantly different, as compared to the selenite control group (p < 0.05)

In the present study a significant decrease (p < 0.05) in the amount of soluble protein and total protein in selenite treated group was observed, as compared to the normal control. Treatment with *Embelica officinalis *(26.19 mg/kg; aqueous extract; p.o.) significantly increased (p < 0.05) the total protein content as well as the soluble protein levels. The increase in the total protein content in the ascorbic acid treated (26.19 mg/kg; p.o.) animals was also found to be more than that of the selenite control. 

*Antioxidant activity of Embelica officinalis*

*(I) Effect of Embelica officinalis on lipid peroxidation (MDA content)*

Rats administered with sodium selenite showed a significant increase (p < 0.05) in the levels of malondialdehyde (Table 1), compared to the normal control. The elevation produced in lipid peroxidation by selenite was decreased significantly (P < 0.05), as a result of *Embelica officinalis *(26.19 mg/kg, aqueous extract; p.o.) administration. The reversal offered by ascorbic acid (26.19 mg/kg) on malondialdehyde level was comparable with the respective normal control values.

*(II) Effect of Embelica officinalis on lens glutathione levels*

Selenite injection significantly decreased (p < 0.05) glutathione (GSH) levels. Treatment with *Embelica officinalis *(26.19 mg/kg; aqueous extract; p.o.) significantly increased (p < 0.05) GSH levels. GSH levels were also increased in the ascorbic acid treated (26.19 mg/kg) animals. 

## Discussion

Cataract was produced in suckling rat pups by an overdose of the essential trace mineral selenium, when injected before completion of the critical maturation period of the lens (approximately 16 days of age). 

It has been hypothesized that these early changes in lens epithelium may result from oxidative damage caused by selenite, possibly due to the critical sulfhydryl groups on molecules such as calcium ATPase or ion channels. Loss of calcium haemostasis could be prevented by antioxidants. 

The significant increase in the protein content (both total and soluble proteins) indicates an improvement in the cataractogenic condition, with *Embelica officinalis *treatment. 

L-Ascorbic acid (pure form) did not increase glutathione levels and nor it could decrease the malondialdehyde levels to a significant extent, as was observed with *Embelica officinalis *treatment. This suggests that cataract inhibition by *Embelica officinalis *is due to some other constituents other than vitamin C.

Some studies indicate that the biological actions, particularly antioxidant activities, of *Embelica officinalis *can not be attributed to ascorbic acid alone (11-13). The potent vitamin C-like anti-oxidative effect of *Embelica officinalis *fruit against reactive oxygen species, was also observed with low molecular weight hydrolysable tannins (14). Four such compounds, emblicanin A, emblicanin B, punigluconin, and pedunculagin have been isolated from *Embelica officinalis *pericarp and their structures have been established (14). Furthermore, Bhattacharya *et al *(12, 13) have demonstrated the antioxidant property of tannoid rich fraction of *Embelica officinalis *in a stress induced oxidative damage model in rat brain. Therefore, it can be concluded that the antioxidant potential of *Embelica officinalis, *as evidenced by a significant decrease in malondialdehyde and a simultaneous increase in lens glutathione levels in the present study, may be attributed to tannoid principle and not fully because of ascorbic acid present in *Embelica officinalis *extract. 

It is now well understood that oxidative stress is one of the important reasons for cataract formation, which in turn is associated with alteration in protein content of lens (i.e. decrease in both total and soluble proteins). The antioxidant property of *Embelica officinalis *was confirmed by an increase in lens glutathione content and a decrease in malondialdehyde content. However, at the same time the improvement in the cataractogenic condition was implicated by an increase in the protein content (both soluble and total protein) within *Embelica officinalis.*
